# Estimation of Rice Aboveground Biomass by Combining Canopy Spectral Reflectance and Unmanned Aerial Vehicle-Based Red Green Blue Imagery Data

**DOI:** 10.3389/fpls.2022.903643

**Published:** 2022-05-27

**Authors:** Zhonglin Wang, Yangming Ma, Ping Chen, Yonggang Yang, Hao Fu, Feng Yang, Muhammad Ali Raza, Changchun Guo, Chuanhai Shu, Yongjian Sun, Zhiyuan Yang, Zongkui Chen, Jun Ma

**Affiliations:** ^1^Rice Cultivation Laboratory, Rice Research Institute, Sichuan Agricultural University, Chengdu, China; ^2^Crop Ecophysiology and Cultivation Key Laboratory of Sichuan Province, Chengdu, China; ^3^Sichuan Engineering Research Center for Crop Strip Intercropping System, Chengdu, China

**Keywords:** aboveground biomass, rice, vegetation indices, wavelet features, texture, unmanned aerial vehicle, machine learning

## Abstract

Estimating the aboveground biomass (AGB) of rice using remotely sensed data is critical for reflecting growth status, predicting grain yield, and indicating carbon stocks in agroecosystems. A combination of multisource remotely sensed data has great potential for providing complementary datasets, improving estimation accuracy, and strengthening precision agricultural insights. Here, we explored the potential to estimate rice AGB by using a combination of spectral vegetation indices and wavelet features (spectral parameters) derived from canopy spectral reflectance and texture features and texture indices (texture parameters) derived from unmanned aerial vehicle (UAV) RGB imagery. This study aimed to evaluate the performance of the combined spectral and texture parameters and improve rice AGB estimation. Correlation analysis was performed to select the potential variables to establish the linear and quadratic regression models. Multivariate analysis (multiple stepwise regression, MSR; partial least square, PLS) and machine learning (random forest, RF) were used to evaluate the estimation performance of spectral parameters, texture parameters, and their combination for rice AGB. The results showed that spectral parameters had better linear and quadratic relationships with AGB than texture parameters. For the multivariate analysis and machine learning algorithm, the MSR, PLS, and RF regression models fitted with spectral parameters (R^2^ values of 0.793, 0.795, and 0.808 for MSR, PLS, and RF, respectively) were more accurate than those fitted with texture parameters (R^2^ values of 0.540, 0.555, and 0.485 for MSR, PLS, and RF, respectively). The MSR, PLS, and RF regression models fitted with a combination of spectral and texture parameters (R^2^ values of 0.809, 0.810, and 0.805, respectively) slightly improved the estimation accuracy of AGB over the use of spectral parameters or texture parameters alone. Additionally, the bior1.3 of wavelet features at 947 nm and scale 2 was used to predict the grain yield and had good accuracy for the quadratic regression model. Therefore, the combined use of canopy spectral reflectance and texture information has great potential for improving the estimation accuracy of rice AGB, which is helpful for rice productivity prediction. Combining multisource remotely sensed data from the ground and UAV technology provides new solutions and ideas for rice biomass acquisition.

## Introduction

Nitrogen is the most critical nutrient for promoting crop growth, increasing crop aboveground biomass (AGB), and improving grain yield. As an important predictor, AGB can reflect crop growth status and gross primary production (Harrell et al., 2011) and is related to grain yield (Wang et al., 2021b). Rapid and accurate assessment of crop AGB is essential for predicting grain yield and improvement of field nitrogen management strategies. The conventional approach for measuring AGB by collecting samples in the field and drying them indoors is destructive, time-consuming, laborious, and prone to human error. In recent years, remotely sensed technology has been successfully used to estimate crop AGB, other physiological parameters, and grain yield and quality. With the development of remotely sensed technology, more remotely sensed sensors and platforms have been developed and applied to agricultural condition monitoring.

Remotely sensed data for estimating rice AGB are acquired from the ground (Cheng et al., 2017), unmanned aerial vehicles (UAV; Jiang et al., 2019), and satellite platforms (Sharifi and Hosseingholizadeh, 2020). Remotely sensed data from the ground with hyperspectral information have received close attention and are widely used to estimate crop AGB because ground-based hyperspectral remote sensing has the advantages of high spectral resolution, continuous wavebands, high efficiency, and objectivity (Li et al., 2016a). Many studies have reported the close relationship between spectral parameters and rice biomass using ground-based hyperspectral remote sensing (Casanova et al., 1998; Gnyp et al., 2014; Kanke et al., 2016; Cheng et al., 2017). Ground-based hyperspectral remote sensing cannot directly observe the growth status of rice, nor can it satisfy the requirements of space–time, and it is difficult to estimate rice biomass and dry matter over large areas. Conversely, satellite platforms have an obvious advantage in monitoring rice growth status and estimating biomass over large areas (Mansaray et al., 2020; Sharifi and Hosseingholizadeh, 2020). A study reported using back-propagation artificial neural network models to estimate the grassland AGB from MODIS satellite imagery with high accuracy (R^2^: 0.75–0.85; Yang et al., 2018). However, the estimation accuracy of crop AGB using satellite imagery data is often influenced by spatial and spectral resolution, cloud cover, and meteorological factors (Wu et al., 2005). In particular, the anticipated accuracy is not achieved for small field areas. Many studies have used UAV-based RGB imagery to overcome these constraints and drawbacks and to estimate rice AGB with high accuracy (Cen et al., 2019; Wan et al., 2020).

Multisource remotely sensed data are acquired to provide more approaches for the accurate, fast, and non-destructive monitoring of crop biomass. Researchers have developed various data-processing methods and mathematical models for remotely sensed data. Canopy spectral reflectance, multispectral imagery, and RGB imagery are often used to extract vegetation indices (VIs) for estimating AGB in maize, rice, and wheat (Jiang et al., 2019; Yue et al., 2019; Ma et al., 2020; Raya-Sereno et al., 2021). Spectral VIs (SVIs), such as the ratio vegetation index (RVI), difference vegetation index (DVI), and normalized difference vegetation index (NDVI), have proven to have close relationships with rice biomass (Gnyp et al., 2014). However, the relationship between remotely sensed data and physiological parameters is related to differences in crop species, remote sensing measurements, and growth conditions. When SVIs of the same wavebands are used in different crop species or growth conditions, crop biomass could be overestimated or underestimated, yielding larger errors. Therefore, extracting accurate wavebands to establish SVIs is necessary to improve the estimation accuracy of crop biomass. SVIs based on complete two-by-two combinations of spectral wavebands were calculated to accurately estimate leaf chlorophyll content (LCC; Wang et al., 2021b), canopy nitrogen content (Wang et al., 2022), leaf area index (Delegido et al., 2015), and grain yield (Rodrigues et al., 2018). Wavelet analysis is a widely utilized spectral analysis tool that uses mother wavelet functions by decomposing raw spectral reflectance data into multiple scales (Mallat, 1989; Cheng et al., 2011). Continuous wavelet transform (CWT) is superior to SVIs in noise and dimension reduction (Cheng et al., 2011). Our studies have confirmed that CWT has better performance than SVIs for estimating LCC (Wang et al., 2020) and carbon-nitrogen content (Chen et al., 2019; Wang et al., 2022). The wavelet coefficient calculated using mother wavelet functions can minimize the interference of the canopy structure and soil background on the spectral reflectance data (Cheng et al., 2012). Previous studies have analyzed the feasibility of wavelet analysis for estimating crop biomass and dry matter content using remotely sensed data (Li et al., 2013; Cheng et al., 2014). Wavelet analysis has been widely used to estimate physiological parameters (Guo et al., 2015; Wang et al., 2016), predict grain yield and protein content (Wang et al., 2021b), and detect weeds and diseases in the field (Zhang et al., 2014).

RGB imagery shows abundant color and texture features with temporal and spatial information, compensating for the defects of ground-hyperspectral remote sensing. However, extracting information from RGB imagery is more complicated than ground-hyperspectral remote sensing. Multiple RGB images obtained from the field are stitched to yield orthophotos and point cloud data, and then the digital number (DN) values and texture features are extracted to monitor the growth status of crops. Two complementary data sources are used simultaneously to improve the estimation accuracy of wheat biomass (Lu et al., 2019). Generally, both RGB-based VIs (RGB-VIs) and texture features derived from RGB imagery are vital variables for estimating crop biomass. The estimation accuracy of crop biomass is gradually considered based on the influence of crop growth differences. The majority of previous studies have attempted to combine RGB-VIs and plant height information to improve the accuracy and have shown the best estimation performance (Bendig et al., 2014; Iqbal et al., 2017; Lu et al., 2019). One study found that coupled plant height and spectral reflectance data correlated with barley biomass (Bendig et al., 2015). Spectral reflectance data reflect the specific features (such as steps, reflection peaks, and absorption valleys) of physiological and biochemical information in crop tissues with a strong correlation. Therefore, establishing a multiple stepwise regression (MSR) model using texture features and SVIs (Yue et al., 2019) is a better approach to estimating crop biomass than plant height (Bendig et al., 2015; Roth and Streit, 2018). Satellite imagery and polarimetric radar data were combined to improve the estimation model of rice biomass on the Sanjiang Plain in Heilongjiang Province, Northeast China (Koppe et al., 2013). The combination of multisource remotely sensed data is receiving extensive attention for estimating the physiological parameters and productivity of crops.

Multivariate analysis and machine learning algorithms have great potential in remotely sensed data mining and crop biomass estimation, with higher accuracy than convenient algorithms (Meyer and Neto, 2008; Mansaray et al., 2020). The MSR model established by a combination of SVIs and texture indices can explain more variability of rice AGB (R^2^ = 0.87) than linear and exponential regression models for the pre-heading stage (Zheng et al., 2019). The random forest (RF) regression model was used to achieve a better prediction result (R^2^ = 0.90) than linear and exponential regression models for rice AGB (Jiang et al., 2019). Few studies have investigated multivariate analysis and machine learning algorithms for estimating rice AGB by combining SVIs and wavelet features (spectral parameters) derived from canopy spectral reflectance and RGB-VIs, texture features and texture indices (texture parameters) derived from RGB imagery. Consequently, this study examines whether RGB-VIs and texture parameters can compete with spectral parameters for estimating rice AGB. We determine whether the combination of canopy spectral reflectance and RGB imagery leads to a more accurate estimation of rice AGB. Last, we evaluate the performance of estimation models of rice AGB established using univariate analysis, multivariate analysis, and machine learning algorithm from canopy spectral reflectance and RGB imagery.

## Materials and Methods

### Field Experimental Details

**Figure 1:**
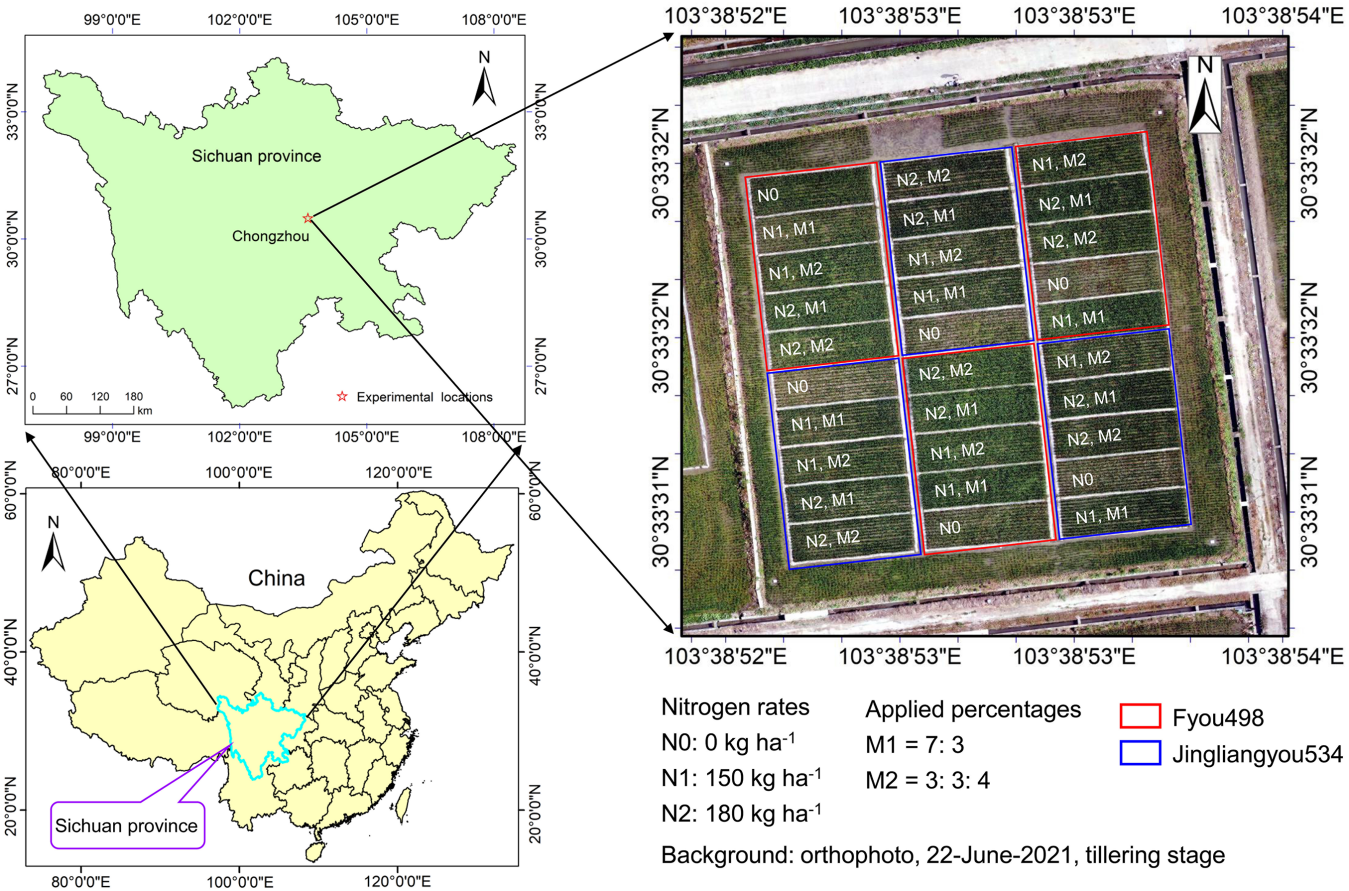
Location of the field experimental site and layout of the field plots with two rice cultivars, three nitrogen rates, and two applied percentages.

The field experiment was conducted in 2021 at the Sichuan Agricultural University Modern Agricultural Research and Development Base in Chongzhou city (30°33′N, 103°38′E, altitude 540 m), Sichuan Province, China (Figure 1). The experimental location is in a subtropical humid monsoon climate zone; the average temperature is 23.7°C, and precipitation is 908.4 mm from May to September during the rice-growing season.

In a randomized complete block design, experimental treatments were carried out with three replications for three nitrogen rates, two nitrogen application percentages, and two cultivars during three growth stages. The nitrogen rates were applied as urea (46.7% N) at N0 (0 kg ha^−1^), N1 (150 kg ha^−1^), and N2 (180 kg ha^−1^). The nitrogen fertilizer was applied in two percentages as follows: i) approximately 70% of urea was applied as basal fertilizer, and another 30% of urea was applied at the tillering stage (M1 = 7: 3); and ii) 30% of urea was applied as basal fertilizer, 30% of urea was applied at the tillering stage, and another 40% of urea was applied at the heading stage (M2 = 3: 3: 4). Two rice cultivars, “Fyou498” with loose type and “Jingliangyou534” with compact type, were sown on April 14, rice seedlings were transplanted with a row space of 0.33 m and plant distance of 0.17 m on May 16. Rice grain was harvested on September 12. A total of 30 plots were used for the experiment, and the individual plot size was 11 × 4 m^2^. The plant density was 1.8 × 10^5^ plants ha^−1^. Other basal fertilizers of phosphorus as calcium superphosphate (12% P_2_O_5_) at 75 kg ha^−1^ and potassium as potassium chloride (60% K_2_O) at 150 kg ha^−1^ were applied to all plots. Weeds and insect populations were controlled with herbicides and pesticides, respectively.

### Data Acquisition

#### Acquisition of RGB Imagery

Acquisition of RGB imagery was performed at the tillering (June 22), booting (July 21), and full-heading (August 03) stages. RGB imagery and canopy spectral reflectance were acquired under clear sky conditions between 10:00 and 14:00 (Beijing local time). We used a UAV platform with four propellers and a visible RGB camera (DJI Mavic 2 Zoom, DJI, Shenzhen, China) to fly over the rice field and evaluate the RGB-VIs and texture features for estimating rice biomass. The detailed specifications of the aircraft, camera, and flight settings are shown in Table 1. The aircraft was flown before the measurement of canopy spectral reflectance to avoid the human campaigns from destroying the canopy status, which would have affected the RGB imagery. According to the settings of camera specifications and flight details, the nine routes were automatically fielded from west to east. Nine images were acquired for each route, for 81 images. The aircraft was always stable during flight, and flight planning was not changed during the whole season. Orthophotos were generated using Agisoft PhotoScan software (Agisoft, LLC., St. Petersburg, Russia) to extract DN values and texture features.

**Table 1:** Basic information on aircraft, cameras, and flight settings.

Aircraft		Camera		Flying details	
Take-off weight	905 g	Camera model	FC2204	Height	30 m
Maximum flying speed	20 m s^−1^	Effective pixels	12 million	Speed	2.2 m s^−1^
Maximum flying height	6,000 m	CCD	1-inch CMOS	Shutter interval	4 s
GNSS	GPS + GLONASS	Angle	85°	Ground resolution	1.1 cm pixel^−1^
		Photo resolution	4,000 × 3,000	Forward overlap	80%
		Bit depth	24	Side overlap	70%
		Aperture	f/2.8		
		Focal length	4.386 mm		
		ISO	ISO-100		
		Exposure	1/640 s		
		Photo format	JEPG (RAW)		

#### Measurement of Canopy Spectral Reflectance

After acquiring RGB imagery, ground-based canopy spectral reflectance data were measured using a field spectroradiometer with a 25° field-of-view fiber optic probe (AvaSpec-2048, Avantes, Apeldoorn, Netherlands). The device has a full spectral range from 350 nm to 2,500 nm, and the sampling intervals are 0.6 nm from 350 nm to 1,100 nm and 6 nm from 1,100 nm to 2,500 nm. The probe was vertically placed from 1 m above the rice canopy and 0.445 m view diameter to obtain spectral information. A 25π m^2^ BaSO_4_ white panel was used to calibrate spectral reflectance before and after vegetation measurement by using three scans each time. Canopy spectral reflectance was measured for three samples in a plot, and average reflectance was recorded by scanning three times for one sample.

#### Measurement of Aboveground Biomass and Grain Yield

After measuring canopy spectral reflectance, rice plants were uprooted and taken to the laboratory to remove the dirt and soil. Then, the roots of the rice plants were cut off and placed in paper bags. Samples were oven-dried at 105°C for 0.5 h and 70°C until constant weight. Subsequently, dry weight (including stems, leaves, and panicles) was weighed and recorded. Rice AGB (kg m^−2^) was calculated as the product of dry weight per plant (kg plant^−1^) and plant density (plant m^−2^; Li et al., 2016b).

Rice grain yield (t ha^−1^) for each plot was harvested individually by manual means at the maturity stage. The collected rice grain was air-dried to a 13.5% moisture level and weighed using an electronic balance.

### Data Analysis

#### Texture Analysis

**Figure 2:**
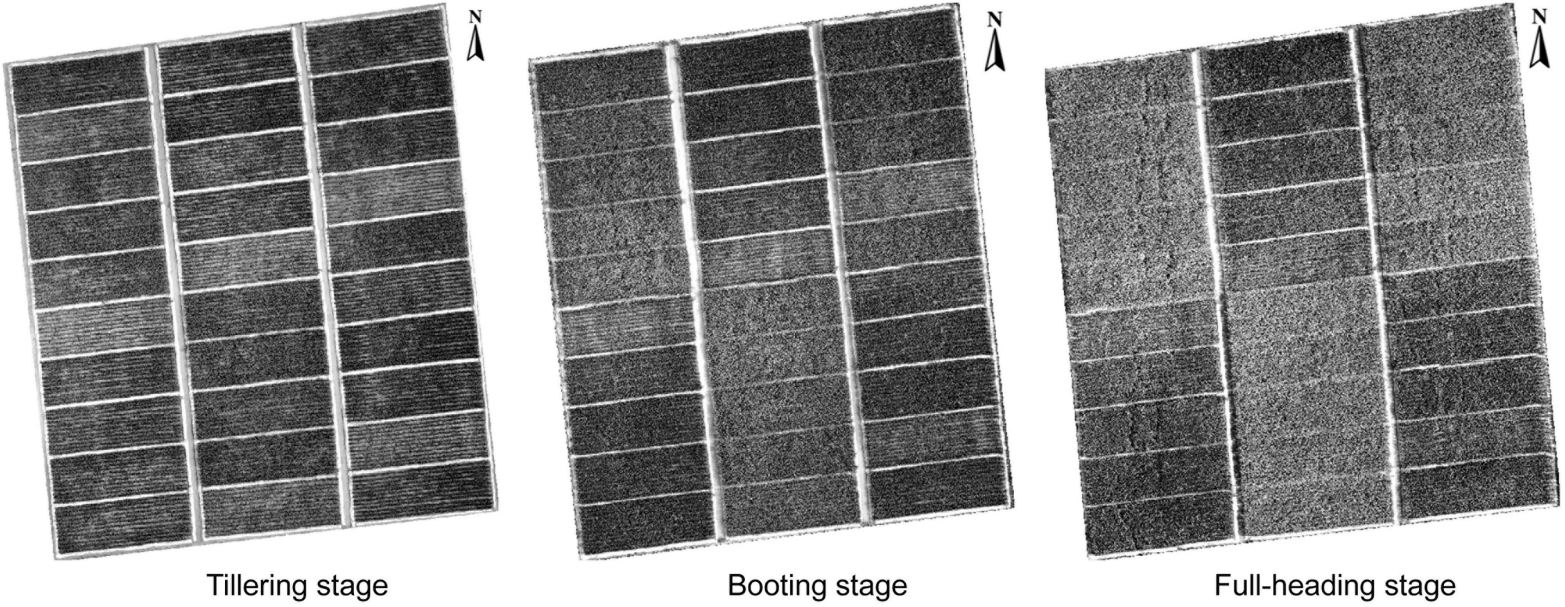
Gray level co-occurrence matrix of rice RGB imagery at the tillering, booting, and full-heading stages.

This study generated a gray level co-occurrence matrix (GLCM) at the tillering, booting, and full-heading stages to analyze the texture features for AGB estimation (Figure 2). Eight texture features were computed in the IDL/ENVI 5.3 environment (Exelis Visual Information Solutions, Boulder, Colorado, United States), including the mean (ME), variance (VAR), homogeneity (HOM), contrast (CON), dissimilarity (DIS), entropy (ENT), second moment (SEM), and correlation (COR). Window size represents detailed texture information and is an important parameter for texture analysis. Appropriate window sizes often contain texture features of the soil background and crop plants (Zheng et al., 2019). Rice was transplanted with a row spacing of 0.33 m and a plant spacing of 0.17 m; thus, texture analysis was performed using the smallest window size of 3 × 3 pixels. The texture features of the red, green, and blue wavebands were calculated separately, and twenty-four features were finally generated. Texture features from RGB imagery were used to evaluate rice AGB.

#### Vegetation Indices and Texture Indices

Orthophotos were processed to extract DN values (including R, G, and B values) using the region of interest tool in IDL/ENVI software. Then DN values were normalized to reduce the illumination effect (Cheng et al., 2001). Five RGB-VIs were studied to correlate with crop physiological parameters and calculated using normalized DN values (i.e., *r*, *g*, and *b*). Based on the RVI, DVI, and NDVI, texture indices were defined as the ratio texture index (TRVI), difference texture index (TDVI), and normalized difference texture index (TNDVI) and produced using complete two-by-two combinations of eight texture features with three wavebands (red, green, and blue wavebands). Texture indices were determined based on a previous study (Zheng et al., 2019). The waveband range of 1,050–2,500 nm was removed because of the exorbitant signal-to-noise ratio. Canopy spectral reflectance with an exorbitant signal-to-noise ratio could affect the sensitive wavebands analysis of rice AGB. Thus, this study used the waveband range of 350–1,050 nm to analyze the relationships between rice AGB and spectral parameters. Eight SVIs of ground-based canopy spectral reflectance were calculated to correlate with rice AGB, including SVIs of complete two-by-two combinations of spectral wavebands within a spectral range of 350–1,050 nm and SVIs of specific spectral wavebands. These indices are defined and listed in Table 2.

**Table 2:** Definition of RGB-VIs and SVIs.

Category	Name	Definition	References
RGB-VIs	Excessive green index	ExG = 2^*^g-*r*-*b*	Woebbecke et al., 1995
	Excessive red index	ExR = (1.4^*^R−*G*)/(*R* + *G* + *B*)	Meyer and Neto, 2008
	Excessive blue index	ExB = (1.4^*^B−*G*)/(*R* + *G* + *B*)	Mao et al., 2003
	Excess green minus excess red	ExGR = ExG−ExR	Camargo Neto, 2004
	Red green blue vegetation index	RGBVI = (*G*^2^−B^*^R)/ (*G*^2^ + B^*^R)	Bendig et al., 2015
Two-by-two combinations of spectral wavebands	Ratio vegetation index	RVI = *R_i_*/*R_j_*	Pearson and Miller, 1972
	Difference vegetation index	DVI = *R_i_*−*R_j_*	Jordan, 1969
	Normalized difference vegetation index	NDVI = (*R_i_*−*R_j_*)/(*R_i_ + R_j_*)	Rouse et al., 1974
Specific spectral wavebands	Red-edge chlorophyll index	CI_rededge_ = *R*_800_/*R*_720_−1	Gitelson et al., 2003
	Renormalized difference vegetation index	RDVI = (*R*_800_−*R*_670_)/(*R*_800_ + *R*_670_)^1/2^	Roujean and Breon, 1995
	Optimized soil adjusted vegetation index	OSAVI = (1 + 0.16)^*^(*R*_800_−*R*_670_)/(*R*_800_ + *R*_670_ + 0.16)	Tremblay et al., 2003
	Transformed chlorophyll absorption reflectance index	TCARI = 3^*^((*R*_700_−*R*_670_)−0.2^*^(*R*_700_-*R*_550_)^*^(*R*_700_/*R*_670_))	Haboudane et al., 2002
	Plant pigment ratio index	PRR = (*R*_550_−*R*_450_)/(*R*_550_ + *R*_450_)	Metternicht, 2003

*R, G, and B represent the DN values of red, green, and blue, respectively; r = R/(R + G + B), g = G/(R + G + B), b = B/(R + G + B); R_i_ and R_j_ indicate the spectral reflectance for wavebands i and j, respectively*.

#### Wavelet Analysis

Five wavelet features, namely daubechies6 (db6), symlets3 (sym3), biorthogonal1.3 (bior1.3), reverse biorthogonal5.5 (rbio5.5), and gaussian3 (gaus3), were executed to transform the spectral reflectance data into wavelet coefficients at a dyadic scale of 1–256 in MATLAB Version 9.2 (MathWorks, Inc., Natick, MA, United States). The definition and equation of wavelet analysis were described in our previous study (Wang et al., 2021b). In this study, spectral reflectance of 350–1,050 nm was used to produce a wavelet coefficient matrix and analyze the correlation between wavelet coefficients and rice AGB on a scale of 1 to 256. Finally, the correlation coefficient matrix diagram, best correlation coefficient (*r*), corresponding waveband, and scale were output to establish the estimation model of rice AGB.

### Model Performance Estimation

Data involving various cultivars, nitrogen rates, and growth stages were integrated to form a comprehensive dataset. The comprehensive dataset was randomly divided into the calibration and validation datasets. 70% of samples were used as the calibration dataset for modeling, and 30% of samples were used as the validation dataset for validating model performance, as shown in Table 3.

**Table 3:** Statistical results of rice AGB and grain yield for calibration and validation datasets.

	AGB (kg m^−2^)		Grain yield (t ha^−1^)	
	Cal. dataset	Val. dataset	Cal. dataset	Val. dataset
Samples	160	68	20	10
Minimum	0.113	0.140	5.839	6.176
Maximum	1.928	2.013	9.204	10.568
Mean	0.885	0.881	8.091	8.352
Standard deviation	0.469	0.490	0.909	1.318
Coefficient of variation (%)	53.0	55.5	11.2	15.8

Three regression methods were selected to evaluate rice AGB, namely, univariate analysis, multivariate analysis, and machine learning algorithm. SVIs, wavelet features, texture features, and texture indices were respectively employed to establish simple linear and quadratic regression models using the calibration dataset for univariate analysis. The MSR and partial least square (PLS) were fitted for multivariate analysis using spectral parameters, texture parameters, and their combination. The MSR can explain the reliability of independent variables and eliminate variables that cause collinearity (Li et al., 2016b). No more than three variables were introduced into the MSR models to avoid overfitting (Zheng et al., 2019). Variables with collinearity and *p* > 0.05 were eliminated for the MSR models.

The PLS regression technique is successfully used to monitor rice biomass (Wang et al., 2021a). The PLS can effectively reduce dimensionality, eliminate the collinearity between variables, and improve the reliability and accuracy of estimation models (Fu et al., 2014). In this study, the PLS regression technique was implemented in MATLAB software, and the modeling results were finally output as regression coefficients, constant, predicted values, and R^2^.

The RF algorithm is an ensemble machine learning algorithm that combines a large set of decision trees to improve the accuracy of classification and regression trees (Mutanga et al., 2012). Two important parameters were adjusted and optimized to achieve the best prediction performance: the number of variables to be tested for each node of tree (*mtry*) and the number of trees (*ntree*). The parameter *mtry* was generally determined from the default value (1/3 of the total number of input variables; Mutanga et al., 2012; Oliveira et al., 2012). In this study, the out-of-bag error rate was calculated to acquire the optimal *mtry*, and the *mtry* with the lowest out-of-bag error rate was selected. Subsequently, we adjusted the parameter *ntree* to achieve the best training results. Finally, *mtry* and *ntree* were determined to operate the RF algorithm using spectral parameters (*mtry* = 1, *ntree* = 1,000), texture parameters (*mtry* = 4, *ntree* = 1,600), and their combinations (*mtry* = 7, *ntree* = 400). The RF algorithm was implemented using the “*randomForest*” package within the R statistical software (R Development Core Team, 2022).

The predictive accuracy of the estimation models was assessed using the R^2^, root mean square error (RMSE), and the ratio of performance to deviation (RPD). The following equations calculated three accuracy metrics:


(1) $$R^{2}=\frac{\overset{n}{\underset{i=1}{\sum }}{\left(y_{i}-y_{i}^{'}\right)}^{2}}{\overset{n}{\underset{i-1}{\sum }}{\left(y_{i}-\bar{y}\right)}^{2}}$$

(2) $$\mathrm{RMSE}=\sqrt{\frac{\overset{n}{\underset{i=1}{\sum }}{\left(y_{i}-y_{i}^{'}\right)}^{2}}{n}}$$

(3) $$\mathrm{RPD}=\frac{Std_{v}}{RMSE_{v}}$$

where $y_{i}$ and $y_{i}^{'}$ are the measured and predicted AGB values for sample i , respectively. $\bar{y}$ is the mean AGB. n is the number of samples for the calibration or validation dataset. $Std_{v}$ and $RMSE_{v}$ are the standard derivation and RMSE of the validation dataset, respectively. Higher R^2^ and lower RMSE values indicate better estimation accuracy for AGB estimation models. The RPD is classified into three levels: RPD ≥ 2 represents good performance, 2 > RPD ≥ 1.4 represents intermediate performance, and RPD < 1.4 represents low performance. The AGB model with the best validation accuracy was selected from all regression models. Rice AGB was used to analyze the relationship with grain yield using the linear model and as a bridge to link remotely sensed data to grain yield (Wang et al., 2021b). The approach indirectly uses remotely sensed data to estimate crop grain yield is more physiologically explanatory.

## Results

### Estimation of Rice Aboveground Biomass Using Spectral Parameters

**Figure 3:**
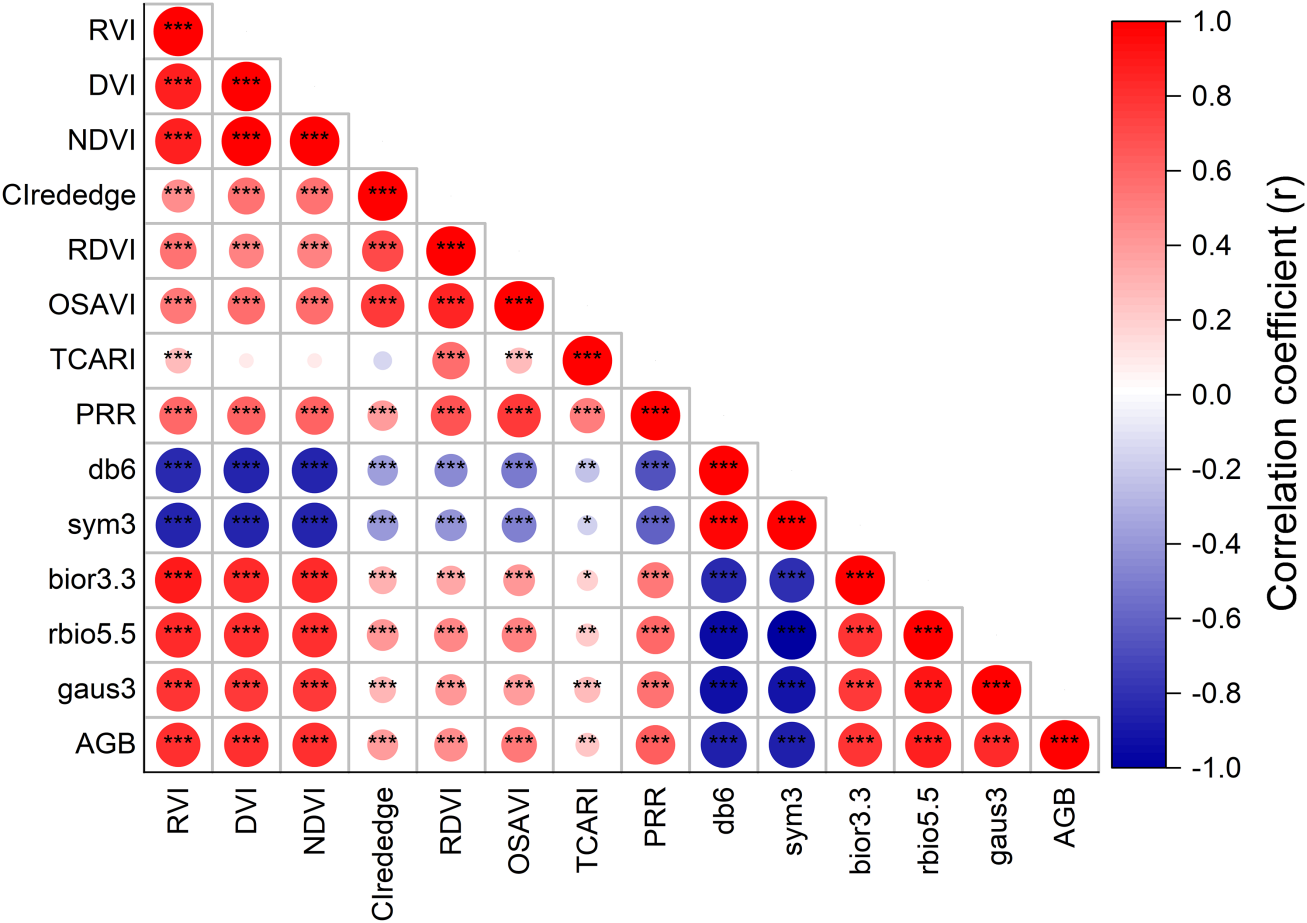
The correlation coefficient between rice AGB and spectral parameters. ^*^ indicates significant correlation at the *p* < 0.05 level, ^**^ indicates the significant correlation at the *p* < 0.01 level, ^***^ indicates the significant correlation at the *p* < 0.001 level.

Figure 3 shows the correlation results for the relationships between rice AGB and spectral parameters. The CI_rededge_, RDVI, OSAVI, TCARI, and PRR of specific spectral wavebands exhibited low positive correlations (*r* < 0.64, *p* < 0.01) with AGB. The correlation coefficient matrices were calculated using complete two-by-two combinations of wavebands (701 × 701 wavebands) for the DVI, RVI, and NDVI (Supplementary Figures S1A–C). The best correlation was selected from the correlation coefficient matrices, and the DVI (952, 947), RVI (775, 784), and NDVI (775, 784) showed better correlation than SVIs of specific spectral wavebands, and the correlation coefficients were 0.811, 0.806, and 0.806, respectively. No differences were found in correlations between the three SVIs. The correlation coefficient matrix diagram illustrates the correlation analysis between wavelet features and rice AGB (Supplementary Figure S2). The wavelet features had a high correlation coefficient (| *r* | > 0.79, *p* < 0.001), and the db6 of the wavelet features had the strongest correlation with AGB (*r* = −0.876, *p* < 0.001) at 469 nm and scale 6. Thus, the DVI (952, 947), RVI (775, 784), and NDVI (775, 784) of SVIs and five wavelet features were adopted to establish the linear and quadratic regression models.

**Figure 4:**
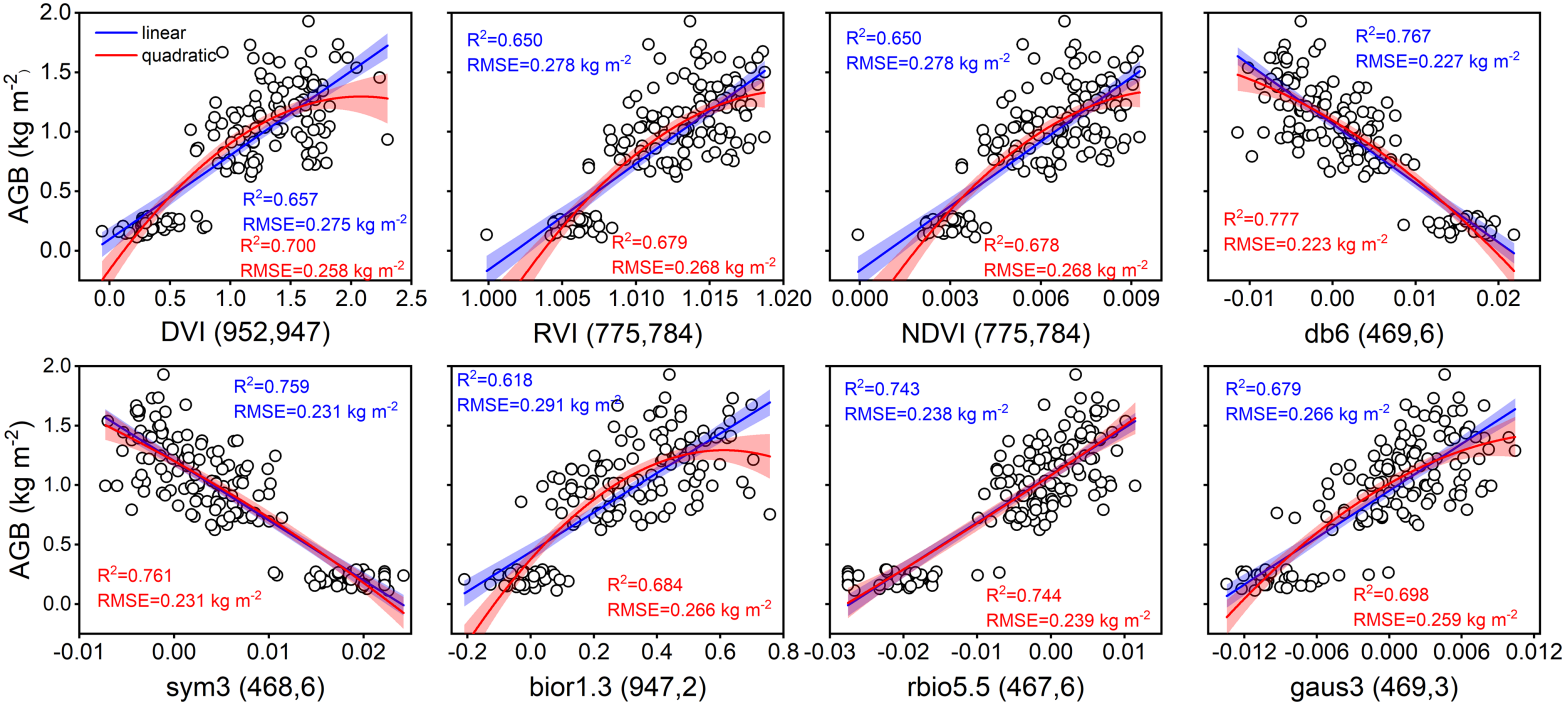
Linear and quadratic regression models using spectral parameters for estimating rice AGB (*n* = 160). The shaded band is the prediction interval at the 95% confidence level. DVI (952, 947) indicates the DVI at wavebands 952 nm and 947 nm. Others are the same as it. db6 (469, 6) indicates the db6 of wavelet features at waveband 469 nm and scale 6. Others are the same as it.

**Figure 5:**
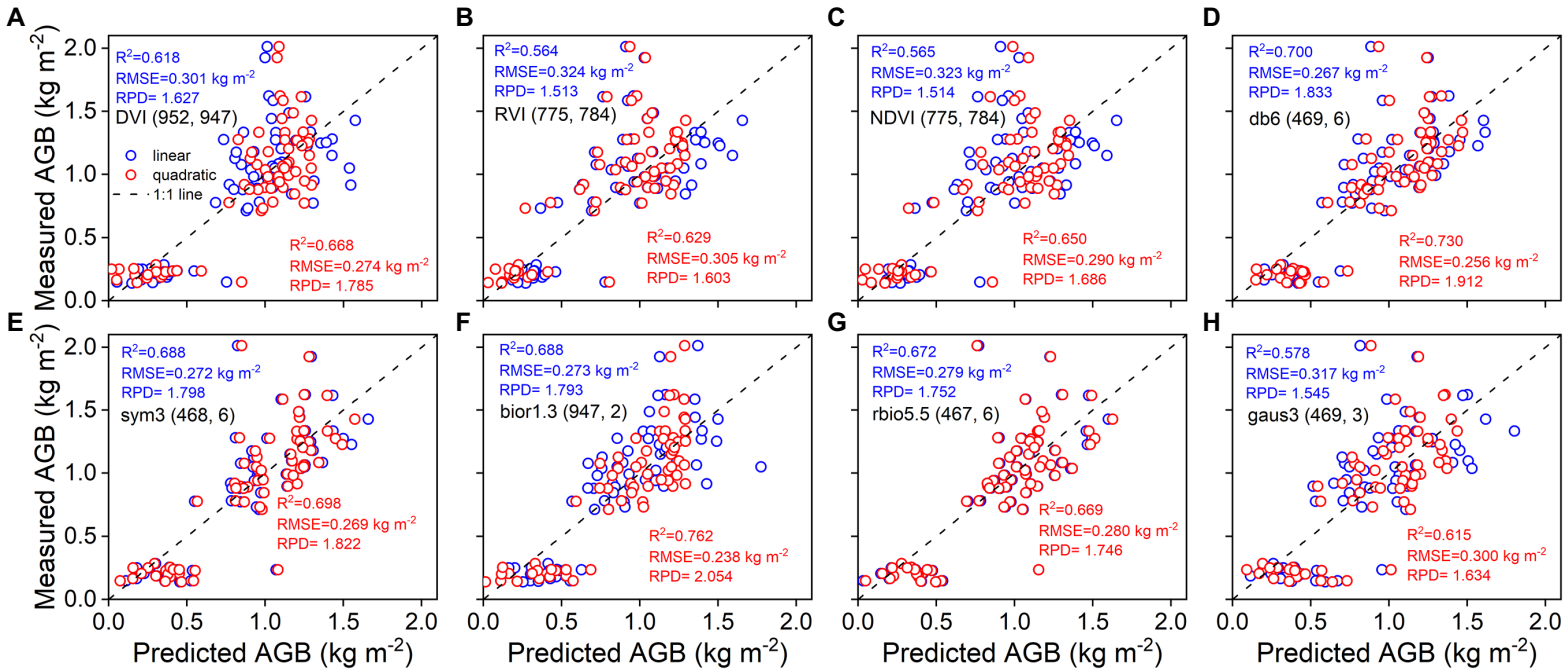
Predicted and measured values of rice AGB with linear and quadratic regression models (*n* = 68). Uppercase letters **(A–H)** are represented in sequence as DVI (952, 947), RVI (775, 784), NDVI (775, 784), db6 (469, 6), sym3 (468, 6), bior1.3 (947, 2), rbio5.5 (467, 6), and gaus3 (469, 3), respectively.

As shown in Figure 4, the accuracy of linear and quadratic regression models is compared to estimate rice AGB using spectral parameters. Generally, the accuracy of the quadratic regression model is superior to that of the linear regression model. The db6 (469, 6) of the wavelet features achieved the best estimation performance for linear (R^2^ = 0.767, RMSE = 0.227 kg m^−2^) and quadratic (R^2^ = 0.777, RMSE = 0.223 kg m^−2^) regression models. Linear and quadratic regression models had proximate curves and similar accuracies for the db6 (469, 6), sym3 (468, 6), and rbio5.5 (467, 6) of the wavelet features. Furthermore, these models were validated with the validation dataset, and the validation accuracy is shown in the scatter plots of 1:1 (Figure 5). The validation results demonstrated that the performance of the SVIs and wavelet features estimates varied with R^2^ values of 0.56–0.67 and 0.58–0.76, RMSE of 0.27–0.32 kg m^−2^ and 0.24–0.32 kg m^−2^, and RPD of 1.51–1.79 and 1.55–2.05, respectively. The wavelet features had a better performance than the SVIs. The quadratic regression model of rice AGB was determined to have the best validation performance (R^2^ = 0.762, RMSE = 0.238 kg m^−2^, RPD = 2.054) using the bior1.3 (947, 2) of the wavelet features.

### Estimation of Rice Aboveground Biomass Using Texture Parameters

**Figure 6:**
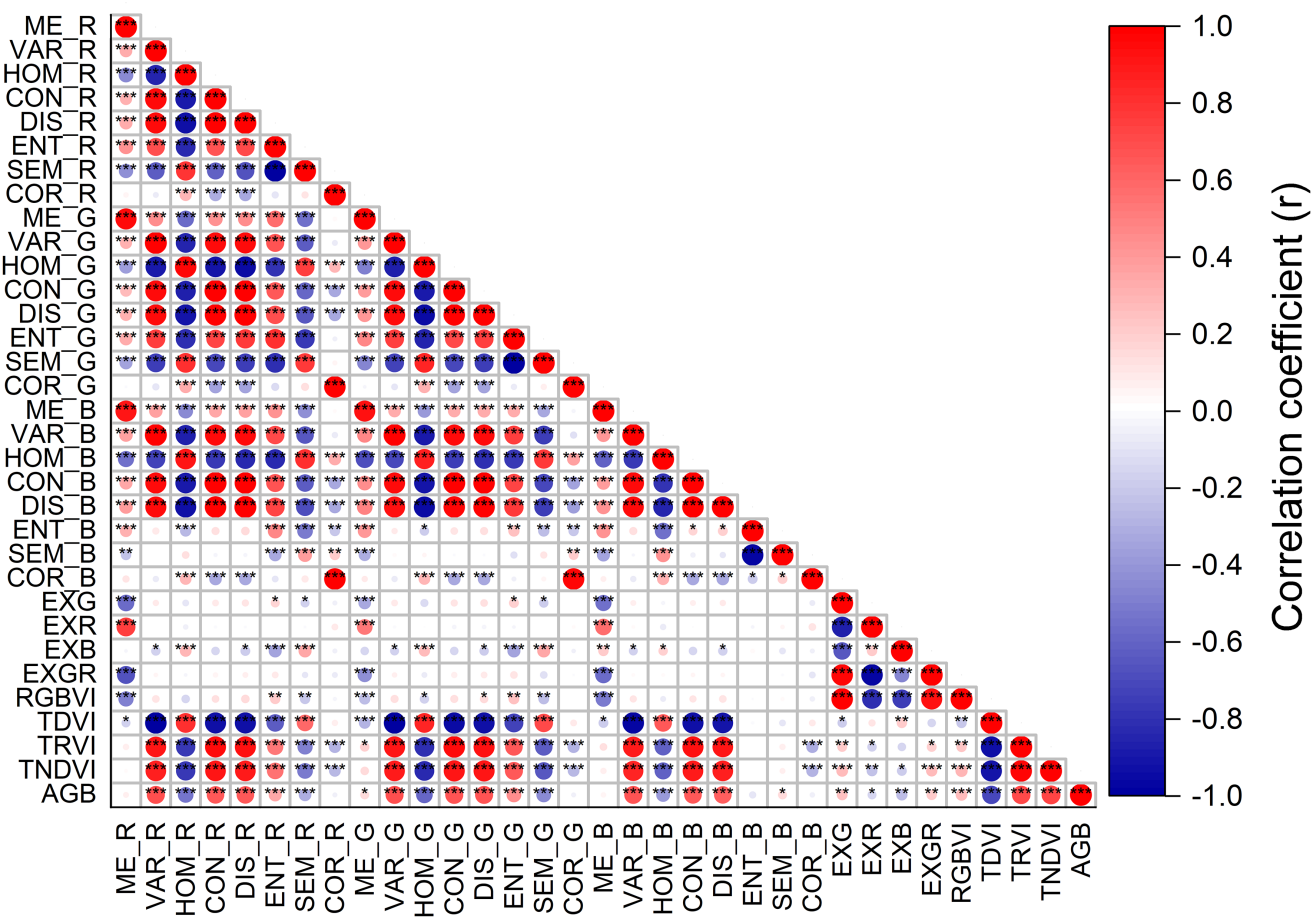
The correlation coefficient between rice AGB and RGB-VIs and texture parameters. ^*^ indicates significant correlation at the *p* < 0.05 level, ^**^ indicates the significant correlation at the *p* < 0.01 level, ^***^ indicates the significant correlation at the *p* < 0.001 level.

The correlation results of rice AGB with RGB-VIs and texture parameters are shown in Figure 6. RGB-VIs exhibited extremely low correlation coefficients with rice AGB, and the maximum correlation coefficient was only 0.277 (*p* < 0.01) with the RGBVI. For the texture features of red, green, and blue wavebands, eleven of twenty-four texture features showed strongly positive or negative correlations with correlation coefficient values from 0.53 to 0.69 (VAR_R, HOM_R, CON_R, DIS_R, VAR_G, HOM_G, CON_G, DIS_G, VAR_B, CON_B, and DIS_B). The strongest correlation was found in VAR_G (*r* = 0.688, *p* < 0.001) with AGB. The correlation coefficient matrices of texture indices were calculated using complete two-by-two combinations of wavebands (24 × 24 wavebands) for the TDVI, TRVI, and TNDVI (Supplementary Figures S1D–F). Texture indices were better correlated with AGB than texture features, with correlation coefficients of −0.708, 0.719, and 0.727 for TDVI (VAR_G, ME_B), TRVI (ME_B, VAR_R), and TNDVI (DIS_B, CON_G), respectively. Thus, RGB-VIs and texture features with low correlation were not used to establish the estimation models of rice AGB.

**Figure 7:**
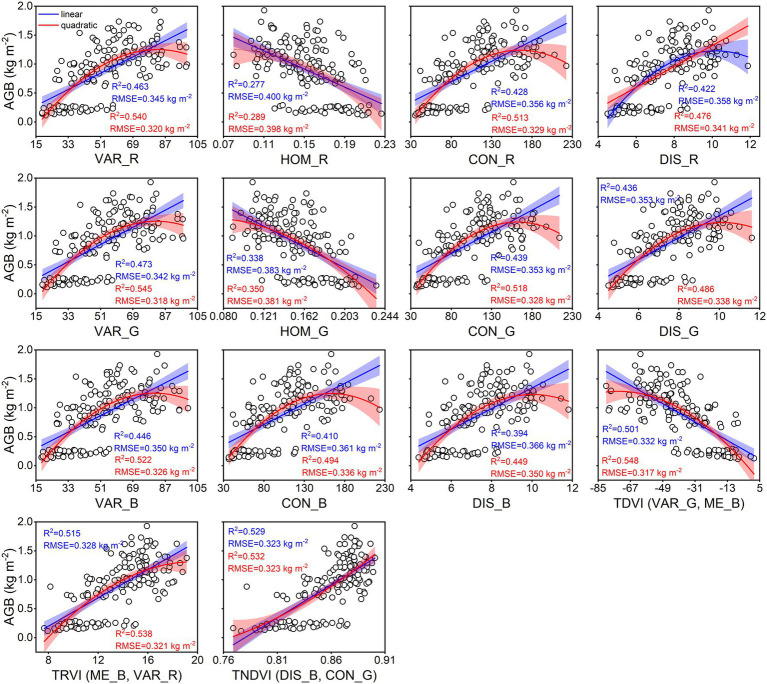
Linear and quadratic regression models using texture parameters for estimating rice AGB (*n* = 160). The shaded band is the prediction interval at the 95% confidence level. TDVI (VAR_G, ME_B) indicates the TDVI at VAR_G and ME_B of texture features. Others are the same as it.

**Figure 8:**
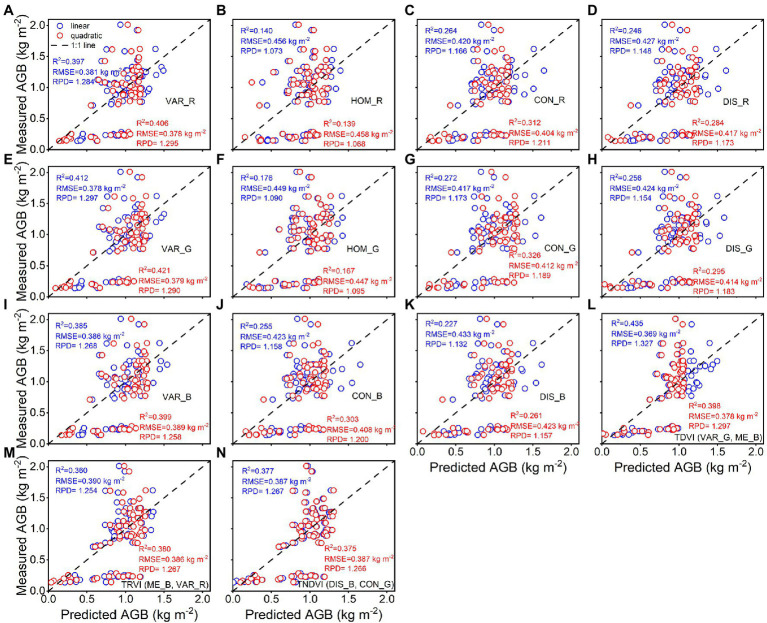
Predicted and measured values of rice AGB with linear and quadratic regression models (*n* = 68). Uppercase letters **(A–N)** are represented in sequence as VAR_R, HOM_R, CON_R, DIS_R, VAR_G, HOM_G, CON_G, DIS_G, VAR_B, CON_B, DIS_B, TDVI, TRVI, and TNDVI, respectively.

The calibration accuracy for linear and quadratic regression models between rice AGB and texture parameters is presented in Figure 7. All estimation models using texture parameters yielded weaker correlations (0.28 < R^2^ < 0.55, 0.32 kg m^−2^ < RMSE <0.40 kg m^−2^) than spectral parameters. The TDVI (VAR_G, ME_B) of texture indices achieved the best calibration performance for quadratic (R^2^ = 0.548, RMSE = 0.317 kg m^−2^) regression models. However, the validation results were unsatisfactory for linear and quadratic regression models using texture parameters (Figure 8), with R^2^ values of 0.14–0.44, RMSE of 0.37–0.46 kg m^−2^, and RPD of 1.07–1.33. These models exhibited low performance (RPD < 1.4) by using texture parameters to estimate rice AGB.

### Estimation of Rice Aboveground Biomass Using Multivariate Analysis and Machine Learning Algorithm

**Figure 9:**
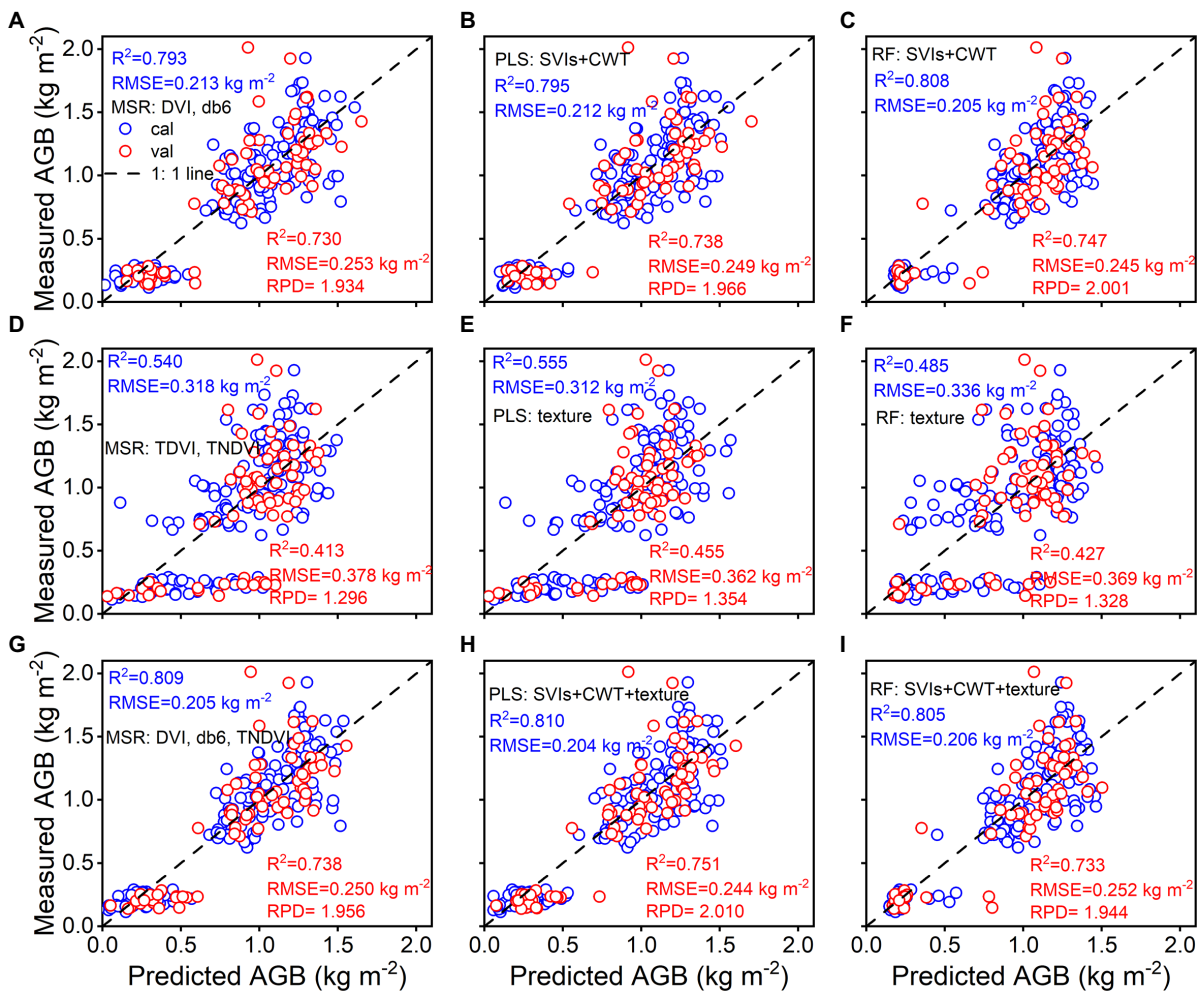
Predicted and measured values of rice AGB with MSR, PLS, and RF regression models from spectral parameters **(A–C)**, texture parameters **(D–F)**, and the combined spectral and texture parameters (**G–I**; cal: *n* = 160, val: *n* = 68).

A combination of spectral parameters, selected texture features, and texture indices was used to investigate rice AGB estimates when using MSR, PLS, and RF techniques. The relationship between the predicted and estimated AGB is shown in Figure 9. The results demonstrated that RF regression using spectral parameters achieved the best calibration (R^2^ = 0.808, RMSE = 0.205 kg m^−2^) and validation (R^2^ = 0.747, RMSE = 0.245 kg m^−2^, RPD = 2.001) accuracy among the three techniques, and MSR and PLS regression had similar accuracy in calibration and validation performance. For selected texture features and texture indices, PLS regression showed better calibration (R^2^ = 0.555, RMSE = 0.312 kg m^−2^) and validation (R^2^ = 0.455, RMSE = 0.362 kg m^−2^, RPD = 1.354) accuracy than MSR and RF regression techniques. When using combined spectral parameters, selected texture features, and texture indices, PLS regression was found to have the best calibration (R^2^ = 0.810, RMSE = 0.204 kg m^−2^) and validation (R^2^ = 0.751, RMSE = 0.244 kg m^−2^, RPD = 2.010) accuracy. Similar accuracy was achieved using MSR and RF regression techniques. The validation accuracy of the three regression techniques was slightly better than that of the linear and quadratic regression models.

### Estimation of Rice Grain Yield Using Remotely Sensed Data

**Figure 10:**
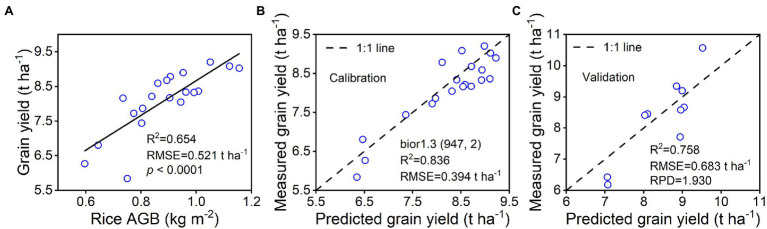
Spectral estimation of rice grain yield by using bior1.3 (947, 2) of wavelet features. **(A)** represents the linear relationship between rice AGB and grain yield (*n* = 20), **(B)** represents the calibration accuracy of the grain yield estimation model (*n* = 20), and **(C)** represents the validation accuracy of predicted and measured grain yield (*n* = 10).

As shown in Figure 10A, a strong linear relationship of grain yield was found with rice AGB (R^2^ = 0.654, RMSE = 0.521 t ha^−1^, *p* < 0.0001). The quadratic regression model established by the bior1.3 (947, 2) of the wavelet features with the highest validation accuracy was selected to investigate the relationship with grain yield. The “AGB-grain yield” linear model was linked with the “bior1.3-AGB” quadratic regression model to generate a spectral estimation model for grain yield. The 1:1 scatter plots of predicted and estimated grain yield are shown for calibration (Figure 10B) and validation (Figure 10C) performance. The results indicated that the bior1.3 (947, 2) of the wavelet features was used to estimate the grain yield with good calibration (R^2^ = 0.836, RMSE = 0.394 t ha^−1^) and validation (R^2^ = 0.758, RMSE = 0.683 t ha^−1^, RPD = 1.930) performance. Ground-based remotely sensed data exhibited a good ability for predicting rice grain yield.

## Discussion

### Relationships Between Rice Aboveground Biomass and Remotely Sensed Data

In this study, the SVIs of specific spectral wavebands from previous studies were found to have low correlations with rice AGB. Generalizing previous studies with our study, the SVIs of specific spectral wavebands were difficult to adapt to the current study (Fu et al., 2014; Bendig et al., 2015; Ren et al., 2018; Wang et al., 2020, 2021b, 2022) because VIs were limited by crop species and the measurement environment of the remotely sensed data. These VIs appeared to be used only for comparison with the new VIs to emphasize the advantages and performance of the new VIs. The sensitive wavebands of rice AGB were found in the red-edge and near-infrared (NIR) regions for the DVI, RVI, NDVI, and bior1.3 of the wavelet features and in the blue wavebands for other wavelet features. According to previous studies, the blue, red-edge, and NIR regions were sensitive to crop biomass (Kanke et al., 2016; Yang et al., 2021). The db6, sym3, and rbio5.5 of the wavelet features showed better calibration performance than the SVIs, which was related to the 467–469 nm of sensitive wavebands and the consistent scale (scale 6). UAV-based RGB imagery is complex and is composed of soil, water, leaves, stems, and panicles (Yue et al., 2019). Although the soil background was effectively classified using a supervised classification method (i.e., RF classifier), DN values were still disturbed by water and soil background, influencing the linear relationship between RGB-VIs and rice AGB. Additionally, the GLCM was used to clearly distinguish crop canopy (dark pixels) and soil background (bright pixels; Haralick et al., 1973), and the correlation with rice AGB was higher than RGB-VIs. The texture imagery showed that the cultivar “Fyou498” had bright pixels with high DN values at the full-heading stage (Figure 2). However, rice crops generally had dark pixels with low DN values, and bright pixels deteriorated the estimation accuracy of rice AGB using texture parameters. The bright pixels might be related to the low chlorophyll content and panicles of cultivar “Fyou498.”

Multivariate analysis and machine learning algorithms have been widely used to predict the AGB and grain yield of crops (Kanke et al., 2016; Cen et al., 2019; Li et al., 2020; Wan et al., 2020; Zhou et al., 2021). Linear and quadratic regression models are the simplest modeling methods used to determine the relationship between two quantitative variables. However, the dependent variable is often related to two or more independent variables. Linear and quadratic regression cannot solve more complex problems and achieve the anticipated prediction ability. In the current study, the MSR, PLS, and RF regression algorithms were able to explain the differences in AGB estimates by multiple variables while improving the prediction accuracy. As the texture features were affected by the water and soil background, the validation accuracy of the MSR, PLS, and RF regression models using texture parameters alone was still unacceptable, as with linear and quadratic regression (RPD < 1.4). Although RGB imagery data have little effective information and are easily affected by complex backgrounds, they cannot eliminate the advantages of RGB imagery data for the monitoring of crop growth status and the estimation of physiological parameters (Li et al., 2016b; Zhou et al., 2020, 2021). The PLS regression algorithm was used to estimate AGB with the best performance by combining spectral and texture parameters (Figure 9H), which explained 2.1%, 1.9%, and 0.2% higher variability than the spectral parameters used and explained 50.0%, 45.9%, and 67.0% higher variability than the texture parameters used for the MSR, PLS, and RF regression algorithms, respectively. Notably, for the three regression algorithms, the difference in estimation accuracy was very small between the spectral parameters and the combination of spectral and texture parameters. RGB imagery provided the texture features with little effective information, and the spectral parameters masked the contribution of texture parameters. It may also be related to the physiological information, canopy structure, and texture information provided by spectral reflectance. Therefore, as important variables, the spectral parameters greatly contribute to the three regression algorithms. In future work, we will further optimize the RGB imagery data to improve the estimation accuracy by identifying and segmenting the background.

### Collinearity and Importance of Variables for the Multivariate Analysis and Machine Learning Algorithm

**Figure 11:**
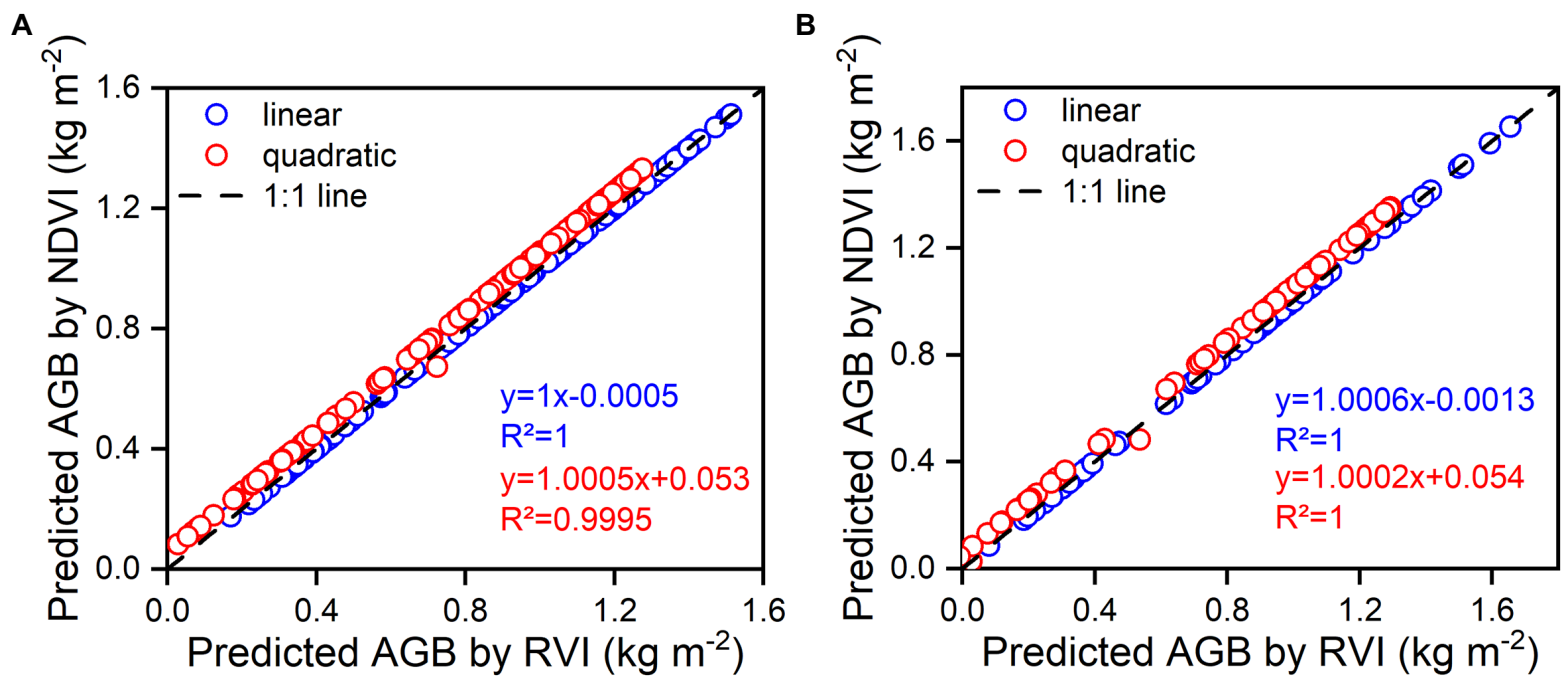
The relationships between predicted values of estimation models using RVI and NDVI for linear and quadratic regression models. **(A)** represents the calibration models, **(B)** represents the validation models.

The DVI, RVI, and NDVI of the complete two-by-two combination of spectral wavebands showed a high correlation with rice AGB. However, linear and quadratic regression models for the RVI and NDVI (Figure 4) exhibited similar scatter distributions and prediction intervals (at the 95% confidence level), resulting in approximate calibration and validation accuracy. The predicted values of the linear and quadratic regression models established by the RVI and NDVI were analyzed (Figure 11), the slope and R^2^ value reached 1 between the predicted values, and the scatter plots were close to the 1:1 line. Figures 3, 11 indicated that the RVI and NDVI had a strong positive correlation (*r* = 1, *p* < 0.001), which also confirmed the strong collinearity of these two VIs. The same wavebands were selected to calculate the RVI, and the NDVI was the main reason for strong collinearity and “perfect” predicted values. Therefore, the relationship between spectral reflectance data and rice AGB, along with the collinearity risk, should be improved.

A parsimonious variable selection method such as MSR is set to no more than three variables to prevent overfitting and collinearity problems (Zheng et al., 2019). When more variables are introduced into the MSR model to estimate rice AGB, collinearity between independent variables should be considered to avoid undermining the stability and reliability of the model. Tolerance and the variance inflation factor (VIF) were employed to assess the collinearity of variables; variables with a tolerance less than 0.1 and a VIF greater than 10 were considered collinear and were not introduced into the MSR model (Dormann et al., 2013). The collinearity diagnosis of the MSR model for estimating rice AGB is shown in Supplementary Table S1. The variables introduced by the MSR model did not have collinearity and achieved a significant level (*p* < 0.05). Some variables were not collinear, but these variables were removed because they did not satisfy the MSR model (*p* > 0.05). For example, the RVI, NDVI, bior1.3, and gaus3 of the wavelet features did not exhibit collinearity but were not employed in the MSR model. The introduction of other variables masks the strong collinearity between the RVI and NDVI. The sym3 and rbio5.5 of the wavelet features and the TRVI always maintained strong collinearity with the other variables. Strong collinear variables will inevitably affect the test accuracy and model application for estimating rice AGB. PLS and RF regression models can accommodate collinearity without deteriorating the predictive performance of rice AGB (Dingstad et al., 2004). Thus, the collinearity of the PLS and RF regression models is not discussed in this study.

**Figure 12:**
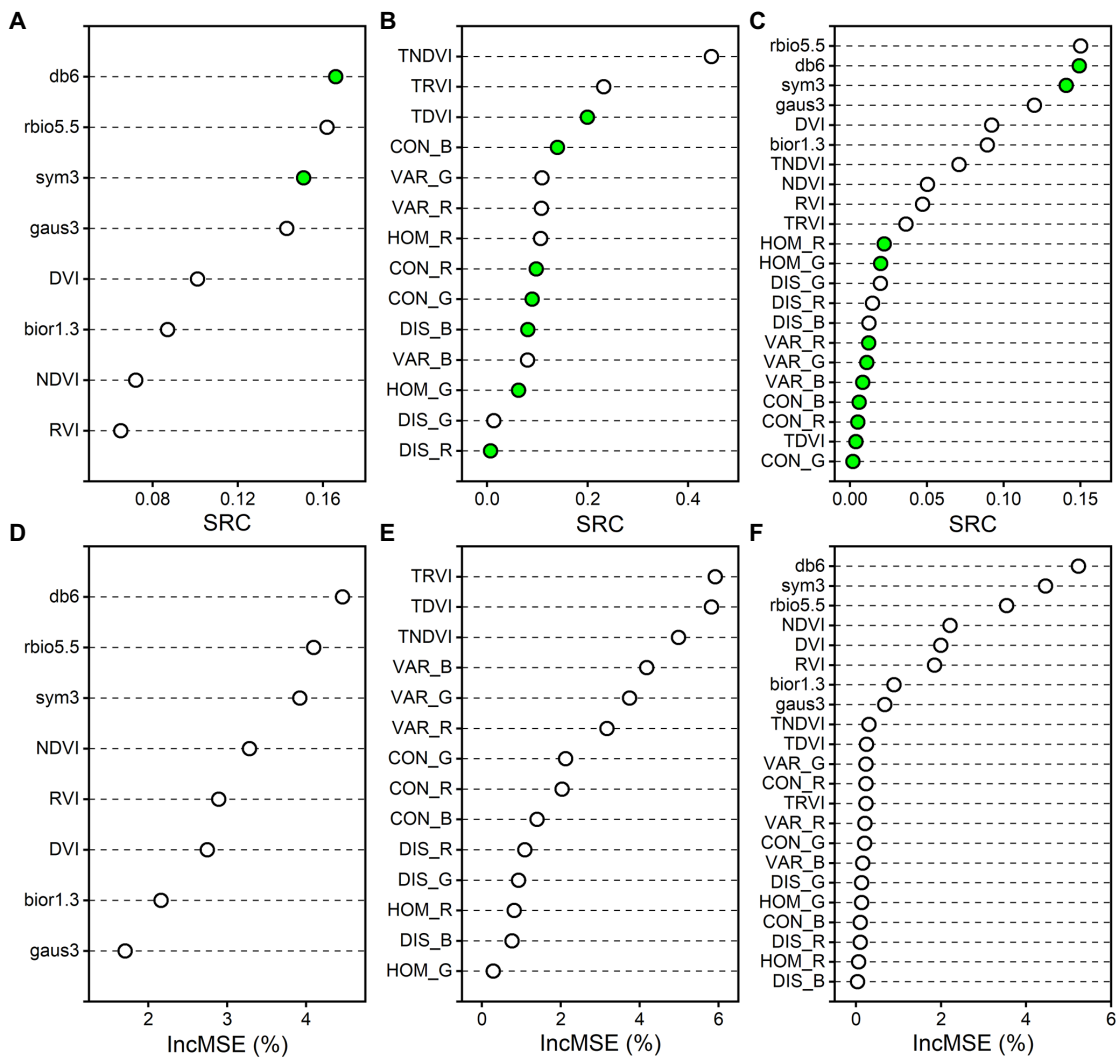
The variable importance measures for the PLS and RF regression models. **(A–C)** indicate the standard regression coefficient (SRC) of PLS regression model for spectral parameters, texture parameters, and the combined spectral and texture parameters, respectively. **(D–F)** indicate the percentage increase in mean square error (IncMSE%) of RF regression model for spectral parameters, texture parameters, and the combined spectral and texture parameters, respectively. Green solid circles indicate absolute values of negative SRC.

The variable importance is supposed to assess the contribution and explanation to rice AGB. The standard regression coefficient (SRC) for the PLS regression model (Prasad et al., 2008) and the percentage increase in mean square error (IncMSE%) for the RF regression model (Oliveira et al., 2012) were used to indicate the variable importance (Figure 12). The larger the absolute value of SRC and IncMSE% is, the greater the influence of the variable on the AGB estimation model. The db6, sym3, and rbio5.5 of the wavelet features had high absolute values of SRC and IncMSE% in the PLS and RF regression models based on spectral parameters and combined spectral and texture parameters. For the correlation analysis between wavelet features and rice AGB, the db6, sym3, and rbio5.5 of the wavelet features were strongly correlated with rice AGB. These three wavelet features were determined to contribute greatly to the estimation of rice AGB. The TDVI, TRVI, and TNDVI of the texture indices significantly contributed to the PLS and RF regression models based on texture parameters. For the combined spectral and texture parameters, the spectral parameters were the main contributions, and the improved estimation accuracy of the MSR and PLS regression models may have been due to the dominant role of spectral parameters. When spectral parameters were coupled with texture parameters, the calibration and validation accuracy of the RF regression model was slightly reduced (Figures 9C,F,I). This may have been because the texture parameters were introduced into the RF algorithm, and the estimation accuracy was weakened for estimating rice AGB. Variables with low contributions tend to be removed. However, the MSR and PLS regression models were slightly improved, reminding us that texture parameter contributions cannot be ignored.

### Sensitivity of Rice Aboveground Biomass

Several studies have demonstrated that AGB data in winter wheat and rice were universally underestimated at the late-reproductive growth stages due to high canopy coverage and plant density (Fu et al., 2014; Kanke et al., 2016; Lu et al., 2019; Zheng et al., 2019). However, samples with high AGB values are not always underestimated (Li et al., 2020; Yang et al., 2021). The underestimation problem can be addressed well by texture parameters and canopy height (Bendig et al., 2015; Yue et al., 2019). The predicted AGB did not suffer from the underestimation problem in this study. Notably, the AGB values derived from the 0–0.5 kg m^−2^ range at the tillering stage were always overestimated (Figures 5, 8, 9). The spectral reflectance and RGB imagery data were not sensitive to rice AGB in the range of 0–0.5 kg m^−2^ (Figures 4, 7). Bare soil and water background during the tillering stage may be the main reasons that interfere with the spectral reflectance and DN values. When the spectral and texture parameters were combined, the predicted AGB values were overestimated, which improved compared to the spectral parameters alone and the texture parameters alone. A study demonstrated that texture information provides the advantage of structural information when spectral information deteriorates biomass estimation accuracy at the heading stage (Lu and Batistella, 2005). The sensitivity of texture parameters at the early vegetative growth stages to vegetation canopy structure remains to be further studied and discussed. Canopy height as an indicator had a suitable and robust relationship with crop biomass and was used to overcome the underestimation problem (Tilly et al., 2015; Li et al., 2016b). We will explore whether canopy height can optimize and improve the overestimation problem of rice AGB.

Previous studies have reported that multivariate analysis and machine learning algorithms, such as MSR (Gab-Sue et al., 2006), PLS (Kawamura et al., 2018), RF (Mariano and Mónica, 2021), and neural networks (Zhou et al., 2021), demonstrate excellent advantages in estimating crop grain yield. However, using machine learning algorithms to predict grain yield is difficult to explain physiologically through physiological parameters and lacks a mechanism. Because AGB is closely related to photosynthetically active radiation and dry matter accumulation in crops, it is a critical predictor of grain yield. SVIs, wavelet features, and texture features were successfully used to estimate crop grain yield (Rodrigues et al., 2018; Maimaitijiang et al., 2020; Wang et al., 2021b). A combination of spectral and texture parameters to predict rice grain yield requires further work. More diverse remotely sensed data are available for improving the estimation models and application areas (Li et al., 2016b). Meanwhile, dry matter is stored in leaves and stems during the vegetative growth stages and then transported at the grain filling stage to form the grain yield. The sensitivity of AGB at the different growth stages was different for grain yield. Therefore, it is essential to seek sensitive stages to predict grain yield using multisource remotely sensed data.

## Conclusion

This study compared the estimation performance of linear, quadratic, MSR, PLS, and RF regression models for rice AGB estimation with spectral parameters (SVIs and wavelet features), texture parameters (texture features and texture indices), and their combination. The results showed that spectral parameters were strongly correlated with rice AGB, and eleven selected texture features and texture indices were found to have significant but weaker correlations. Spectral parameters were superior to texture parameters in estimating rice AGB for the linear and quadratic regression models. For the MSR, PLS, and RF regression models, a combination of spectral and texture parameters slightly improved estimation performance over the use of spectral parameters or texture parameters alone. Combined remotely sensed data may help overcome the overestimation of rice AGB in the range of 0–0.5 kg m^−2^. At the same time, rice grain yield can be predicted well with bior1.3 of the wavelet features. However, this study was limited to one growing season and an area with few datasets. Future work will further optimize texture information and combine spectral reflectance to improve the estimation accuracy of rice AGB and grain yield. More years and growth areas should be examined to test the stability and reliability of the estimation models.

## Data Availability Statement

The original contributions presented in the study are included in the article/Supplementary Material, further inquiries can be directed to the corresponding author.

## Author Contributions

JM and ZW designed the research. JM supervised the research. ZW, YM, PC, YY, and HF performed the experimental work and acquired the data. ZW wrote the first manuscript. ZW, YM, and PC analyzed the data. ZW, FY, MR, CG, and CS revised the manuscript. FY, YS, ZY, and ZC provided helpful suggestions for the experimental work. All authors contributed to the article and approved the submitted version.

## Funding

The work was supported by the Rice Breeding Project Foundation of the Sichuan Provincial Science and Technology Department (grant no. 2021YFYZ0005) and the National Modern Agricultural Industry Technology System Sichuan Innovative Rice Innovation Team Project (sccxtd-2021-01).

## Conflict of Interest

The authors declare that the research was conducted in the absence of any commercial or financial relationships that could be construed as a potential conflict of interest.

## Publisher’s Note

All claims expressed in this article are solely those of the authors and do not necessarily represent those of their affiliated organizations, or those of the publisher, the editors and the reviewers. Any product that may be evaluated in this article, or claim that may be made by its manufacturer, is not guaranteed or endorsed by the publisher.

## Supplementary Material

The Supplementary Material for this article can be found online at: https://www.frontiersin.org/articles/10.3389/fpls.2022.903643/full#supplementary-material
