# Supplementary material for: Estimation of Rice Aboveground Biomass by Combining Canopy Spectral Reflectance and Unmanned Aerial Vehicle-Based Red Green Blue Imagery Data
Source: Front Plant Sci. 2022 May 27;13:903643. doi: 10.3389/fpls.2022.903643 (PMC9197132; doi:10.3389/fpls.2022.903643)
Supplement: Supplementary file 1 [file Table_1.DOCX]

Supplementary Material

**Table S1** Collinearity statistics of multiple stepwise regression with SVIs, wavelet features, texture features, and texture indices.

| **Data source** | **Variables** | **SRC** | ***p values*** | **Tolerance** | **VIF** |
| --- | --- | --- | --- | --- | --- |
| SVIs, wavelet features | **DVI** | **0.262** | **0.000** | **0.311** | **3.212** |
|  | RVI | 0.064 | 0.436 | 0.196 | 5.106 |
|  | NDVI | 0.075 | 0.331 | 0.224 | 4.467 |
|  | **db6** | **-0.661** | **0.000** | **0.311** | **3.212** |
|  | sym3 | -0.172 | 0.287 | 0.051 | 19.707 |
|  | rbio5.5 | 0.166 | 0.159 | 0.095 | 10.479 |
|  | bior1.3 | 0.021 | 0.807 | 0.179 | 5.589 |
|  | gaus3 | 0.030 | 0.771 | 0.125 | 7.984 |
| Texture features, texture indices | VAR_R | -0.154 | 0.613 | 0.032 | 31.496 |
|  | HOM_R | 0.177 | 0.057 | 0.339 | 2.947 |
|  | CON_R | -0.286 | 0.079 | 0.111 | 9.046 |
|  | DIS_R | -0.263 | 0.088 | 0.123 | 8.104 |
|  | VAR_G | -0.260 | 0.497 | 0.020 | 49.762 |
|  | HOM_G | 0.162 | 0.125 | 0.264 | 3.794 |
|  | CON_G | -0.329 | 0.060 | 0.096 | 10.466 |
|  | DIS_G | -0.300 | 0.075 | 0.103 | 9.715 |
|  | VAR_B | -0.138 | 0.571 | 0.050 | 20.100 |
|  | CON_B | -0.264 | 0.076 | 0.133 | 7.538 |
|  | DIS_B | -0.242 | 0.074 | 0.159 | 6.291 |
|  | **TDVI** | **-0.266** | **0.047** | **0.166** | **6.020** |
|  | TRVI | -0.185 | 0.556 | 0.030 | 33.275 |
|  | **TNDVI** | **0.484** | **0.000** | **0.166** | **6.020** |
| SVIs, wavelet features, texture features, and texture indices | **DVI** | **0.230** | **0.000** | **0.305** | **3.276** |
|  | RVI | 0.032 | 0.693 | 0.193 | 5.176 |
|  | NDVI | 0.037 | 0.623 | 0.219 | 4.564 |
|  | **db6** | **-0.559** | **0.000** | **0.258** | **3.871** |
|  | sym3 | -0.141 | 0.366 | 0.051 | 19.769 |
|  | rbio5.5 | 0.133 | 0.244 | 0.095 | 10.552 |
|  | bior1.3 | 0.061 | 0.468 | 0.176 | 5.686 |
|  | gaus3 | 0.015 | 0.878 | 0.125 | 7.998 |
|  | VAR_R | -0.033 | 0.658 | 0.221 | 4.519 |
|  | HOM_R | 0.008 | 0.887 | 0.389 | 2.570 |
|  | CON_R | -0.035 | 0.667 | 0.183 | 5.470 |
|  | DIS_R | -0.015 | 0.853 | 0.188 | 5.321 |
|  | VAR_G | -0.037 | 0.632 | 0.202 | 4.963 |
|  | HOM_G | 0.012 | 0.848 | 0.316 | 3.170 |
|  | CON_G | -0.040 | 0.650 | 0.159 | 6.302 |
|  | DIS_G | -0.012 | 0.889 | 0.160 | 6.252 |
|  | VAR_B | -0.022 | 0.759 | 0.252 | 3.968 |
|  | CON_B | -0.028 | 0.712 | 0.212 | 4.716 |
|  | DIS_B | -0.012 | 0.872 | 0.229 | 4.361 |
|  | TDVI | 0.033 | 0.711 | 0.157 | 6.354 |
|  | TRVI | -0.075 | 0.668 | 0.040 | 24.945 |
|  | **TNDVI** | **0.180** | **0.000** | **0.484** | **2.065** |

Bold indicates variables that were introduced into the multiple stepwise regression models, SRC is the standardized regression coefficient, and VIF is the variance inflation factor.

**Figure S1**. Correlation coefficient matrix of RVI, DVI, and NDVI for spectral **(A-C)** and texture **(D-F)** vegetation indices. RVI (775, 784) indicates the RVI at wavebands 775 and 784 nm. Others are the same as it. 1-8 (9-16 and 17-24) indicate ME, VAR, HOM, CON, DIS, ENT, SEM, and COR of red (green and blue) waveband for texture features, respectively.

**Figure. S2**. Correlation coefficient matrix between rice AGB and wavelet features. The db6 (469, 6) indicates the db6 of wavelet features at waveband 469 nm and scale 6. Others are the same as it.
